# Functional Virtual Flow Cytometry: A Visual Analytic Approach for Characterizing Single-Cell Gene Expression Patterns

**DOI:** 10.1155/2017/3035481

**Published:** 2017-07-17

**Authors:** Zhi Han, Travis Johnson, Jie Zhang, Xuan Zhang, Kun Huang

**Affiliations:** ^1^College of Software, Nankai University, Tianjin, China; ^2^Department of Biomedical Informatics, The Ohio State University, Columbus, OH, USA; ^3^The CCC Biomedical Informatics Shared Resource, The Ohio State University, Columbus, OH, USA

## Abstract

We presented a novel workflow for detecting distribution patterns in cell populations based on single-cell transcriptome study. With the fast adoption of single-cell analysis, a challenge to researchers is how to effectively extract gene features to meaningfully separate the cell population. Considering that coexpressed genes are often functionally or structurally related and the number of coexpressed modules is much smaller than the number of genes, our workflow uses gene coexpression modules as features instead of individual genes. Thus, when the coexpressed modules are summarized into eigengenes, not only can we interactively explore the distribution of cells but also we can promptly interpret the gene features. The interactive visualization is aided by a novel application of spatial statistical analysis to the scatter plots using a clustering index parameter. This parameter helps to highlight interesting 2D patterns in the scatter plot matrix (SPLOM). We demonstrated the effectiveness of the workflow using two large single-cell studies. In the Allen Brain scRNA-seq dataset, the visual analytics suggested a new hypothesis such as the involvement of glutamate metabolism in the separation of the brain cells. In a large glioblastoma study, a sample with a unique cell migration related signature was identified.

## 1. Background

Single-cell RNA sequencing (scRNA-seq) is becoming a powerful tool for studying heterogeneity and subtypes in cell populations. Many bioinformatics and computational tools have been developed to visualize, cluster, and categorize the cells based on their expression profiles [1, 2]. Different algorithmic approaches such as principal component analysis (PCA) or multidimensional scaling (MDS) [3], nonnegative matrix factorization [4], minimum spanning tree (MST) [5, 6], latent variable modeling [7], diffusion map [8, 9], and spline models [10] have all been applied and implemented for such purposes. Moreover, it has been shown that often the cells in a population do not always form “clusters.” Instead, the cells form a continuous distribution over the space of featured genes and gene signatures [1]. Therefore, it is often of great interest to identify the interesting distribution patterns (e.g., wishbone pattern and bifurcation) which often imply important biological processes such as stem cell differentiation as well as the gene signatures that can be used to reveal such patterns.

However, this effort often leads to a “chicken-and-egg” situation. Since the patterns may not always be readily perceivable from whole genome data, methods such as PCA and MDS may not always be effective. Therefore, it often ends up in an iterative process and a subjective selection of genes of interests. Another commonly adopted workflow is to first cluster the cells based on their expression profiles and identify “gene signatures” that differentiate the clusters followed by enrichment analysis on these signature genes for potential biological functions or processes involved in the separation of the cells. Since there could be many genes involved in differential analysis, the functional enrichment signals can be diluted.

In this paper, we propose a visual analytic workflow called functional virtual flow cytometry (FVFC) for identifying functional gene groups that can effectively separate the cells using scRNA-seq data. We specifically take advantage of gene coexpression network analysis (GCNA). GCNA aims to identify modules of genes with similar expression profiles. It has been well known that the coexpressed genes often are functionally or structurally related [11–16]. Therefore, instead of surveying all the genes, by focusing on the coexpressed gene clusters, we can directly study the cells based on functional gene groups with increased statistical power [17].

Our method is innovative in the following ways. First, it focuses on the gene modules with clear functional relationships (coexpression) and thus greatly enhances the statistical power. Secondly, only the gene modules that are “informative” among the single cells are used. Specifically we focus on the modules that show bimodal or multimodal distributions among the cells to ensure separation power of the genes on the cell population. Thirdly, we apply spatial statistical methods to detect combinations of gene modules that lead to interesting spatial patterns or separation of the cells and thus identify the gene signatures associated with the underlying biological processes. Last but not least, instead of developing this workflow as an “algorithm,” we implement it as a visual analytic workflow, allowing the researchers to interactively select gene modules and cell distribution patterns of interest for further investigation. To this end, we take advantage of the SPLOM combined with various visual cues derived from spatial statistical calculation. We demonstrate our workflow using two large single-cell studies on brain and cancer, respectively.

## 2. Methods

### 2.1. Workflow

**Figure 1:**
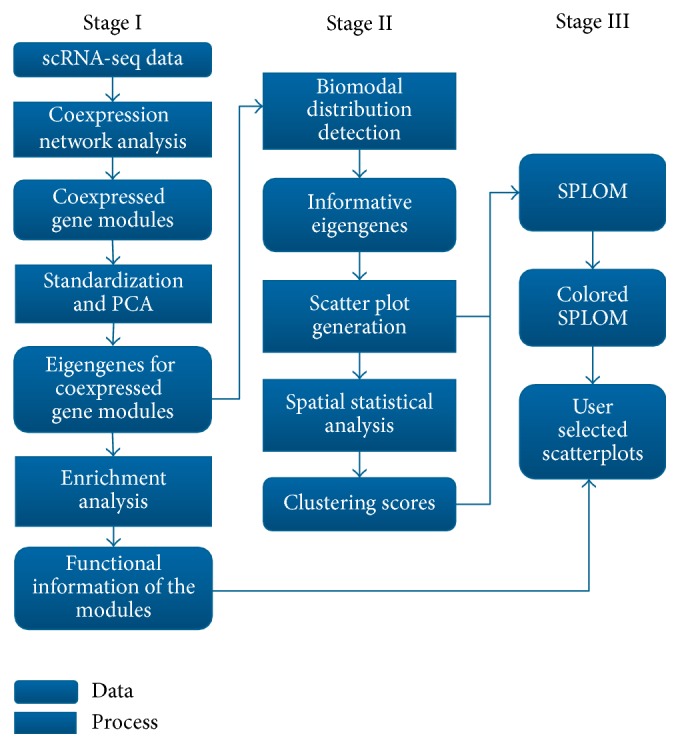
The workflow of the functional virtual flow cytometry system.

Figure 1 outlines the workflow of our approach that contains three stages. Given a set of processed scRNA-seq data, the first stage carries out the coexpression network analysis and summarization of each network module into a single “eigengene” as well as enrichment analysis to determine the function or structural relationships for each module. The second stage analyzes each eigengene to select the ones with more information content, in particular, the bimodal ones. Then scatterplots are generated for every pair of informative eigengenes. The scatterplots are further analyzed using spatial statistical parameters to determine if they form interesting patterns, specifically if there is clustering or clumping in the scatterplot, implying potential relationships between the two gene modules associated with the two eigengenes. In the final stage, the scatterplots are colored based on the spatial statistical parameters and interesting patterns are further examined with their functional relevance. Overall, this workflow provides an intuitive visual analytic approach for researchers to quickly explore the relationships among functional gene groups in single-cell populations. The details of the steps in the workflow are discussed in the following sections.

### 2.2. Weighted Gene Coexpression Network Analysis

**Figure 2:**
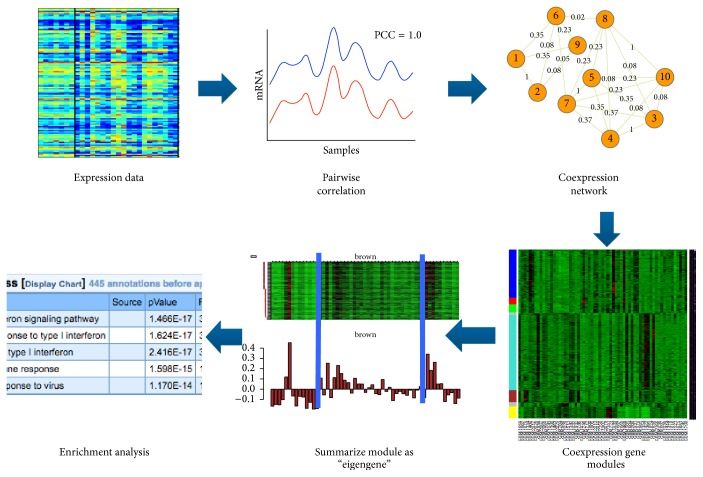
Workflow for weighted GCNA and eigengene calculation.

The first stage in Figure 1 is to carry out gene coexpression network analysis. The detailed workflow for this stage is illustrated in Figure 2. Given a set of *M* genes and their expression levels over *N* cells, the gene expression profile can be expressed with a matrix

(1) $$\begin{array}{c} G=\left[\begin{array}{ccc} g_{11} & \cdots & g_{1N}\\ \vdots & \ddots & \vdots \\ g_{M1} & \cdots & g_{MN} \end{array}\right]=\left[\begin{array}{c} g_{1}\\ \vdots \\ g_{M} \end{array}\right], \end{array}$$

where the *N*-dimensional row vector $g_{i}=\left[\begin{array}{ccc} g_{i1} & \cdots & g_{iN} \end{array}\right]$ is the expression profile for the *i*th gene across the samples (*i* = 1,2,…, *N*). Then the pairwise correlation matrix *C* can be represented by

(2) $$\begin{array}{c} C=\left[\begin{array}{ccc} c_{11} & \cdots & c_{1M}\\ \vdots & \ddots & \vdots \\ c_{M1} & \cdots & c_{MM} \end{array}\right], \end{array}$$

where *c*_*ij*_ is the correlation coefficient between *i*th gene vector **g**_*i*_ and *j*th gene vector **g**_*j*_. In our experiment, we use Spearman rank correlation coefficients in the pairwise correlation matrix since Gaussian distribution cannot be assumed for RNA-seq data as required by Pearson correlation.

After the correlation matrix was computed, we apply a recently developed algorithm called Normalized lmQCM [15]. Compared to widely adopted gene coexpression network analysis software package WGCNA [18], this algorithm takes a network mining approach allowing overlaps between modules and also is guaranteed to have a lower bound on the density of the detected modules. The output of algorithm lmQCM is a set of gene modules  **M**_1_, **M**_2_,…, **M**_*L*_, where each module *M*_*k*_ = {*i*_1_, *i*_2_,…, *i*_*N*_*k*__} is composed of a group of *N*_*k*_ coexpressed genes. The number of modules *L* and the sizes of the modules are determined by the four parameters of the lmQCM algorithm. While detailed choice of parameters was discussed in [15], the most important parameter is **γ**, which is the threshold for the weight of the first edge of any module and thus controls the number of modules. Usually we choose **γ** to ensure that the maximum size of a module is not too large (i.e., less than 500 genes). In addition, we focus on gene modules with at least 10 genes so that meaningful functional enrichment analysis can be applied.

For each gene module detected by lmQCM, *M*_*k*_ can be represented by a gene expression matrix. If we want to compare one gene module against another, it is advantageous to take only a representative of that module rather than taking all the genes. We use PCA to reduce the gene module data meaningfully and take the first principal component as a summary of that module. This first principal component is called “eigengene” in this context. Computationally, we take the submatrix of **G** for *M*_*k*_ as

(3) $$\begin{array}{c} G_{k}=\left[\begin{array}{c} g_{i_{1}}\\ \vdots \\ g_{i_{N_{k}}} \end{array}\right]\in R^{N_{k}\times N}. \end{array}$$

**G**_**k**_ is centralized and standardized as **G**_**k**_′ such that for each row the mean is zero and the norm is one. Let **G**_**k**_′ = **USV**^**T**^ be the singular value decomposition of **G**_**k**_′. Then the first column of **V** (denoted as **v**_1_) is the “eigengene” for **M**_**k**_ up to a sign since **V** is an orthonormal matrix whose determinant is 1 or −1. Since the eigengene **w**_**k**_ should reflect the directions of the majority of genes in **G**_**k**_, its projection on the majority of the genes should be positive. Thus, if ∑sgn⁡(**G**_**k**_**v**_1_) < 0, then **w**_**k**_ = **** − **v**_1_; otherwise, **w**_**k**_ = **v**_1_. So each gene module detected by lmQCM corresponds to one “eigengene.”

For the reported modules, enrichment analyses are carried out using NIH DAVID (https://david.ncifcrf.gov/) [19] and TOPPGene (https://toppgene.cchmc.org/enrichment.jsp) [20].

### 2.3. Identify Eigengenes with Bimodal or Long Tail Distribution

Before exploring pairwise relationships between gene modules with eigengenes, we identify and keep eigengenes which are “informative,” that is, eigengenes whose distribution follows a bimodal or long tail distribution. Therefore, eigengenes with unimodal distribution, especially the ones with narrow sharp peak-shaped distribution, will be filtered out. To differentiate unimodal distribution with bimodal or long tail distributions, metrics such as Kurtosis, second central differences, and likelihood ratio are adopted [21–23]. Specifically, Kurtosis is a measure of the “tailedness” of the probability distribution of a real-valued random variable [24]. Here we use Kurtosis as a measure to filter whether the histogram of a given eigengene has a very narrow sharp peak distribution. For each eigengene vector **w**_**k**_, first the histogram of the vector is computed and then Kurtosis of the histogram distribution is computed as

(4) $$\begin{array}{c} \mathrm{Kurt}\left(\;w_{k}\;\right)=\frac{E\left[{\left(w_{k}-\mu \right)}^{4}\right]}{{\left(E\left[{\left(w_{k}-\mu \right)}^{2}\right]\right)}^{2}}, \end{array}$$

where *μ* is mean of **w**_**k**_. In [24], the Kurtosis value between 3 and 9 show peakness of the distribution while higher values imply sharper peak-shaped distribution. In this paper, we set the threshold for Kurtosis as user defined parameter. If Kurtosis value of histogram for a given eigengene is smaller than a given threshold, then eigengene will be kept.

### 2.4. Spatial Statistical Analysis of the 2D Scatterplot Using the Nearest Neighbor Distribution

In order to find the relationship between two coexpressed gene modules, we generate pairwise scatter plots for all pairs of eigengene vectors in a 2D space. For two given eigengene vectors *e*_*i*_ = [*e*_*i*1_, *e*_*i*2_,…, *e*_*iN*_] and *e*_*j*_ = [*e*_*j*1_, *e*_*j*2_,…, *e*_*jN*_], scatter plot is the points with coordinates (*e*_*i*1_, *e*_*j*1_), (*e*_*i*2_, *e*_*j*2_),…, (*e*_*iN*_, *e*_*jN*_) in the 2D space. Then we use the nearest neighbor distance (NND) to analyze the pattern. NND for a data point is the distance to its closest neighbor. It is a spatial statistical parameter effectively used for detecting cell patterns in the space [25, 26]. Define $\overset{-}{d_{0}}$ as the mean NND for all the points. Then we make 100 random simulations, each time the same number of points is created in the same region covering (*e*_*i*1_, *e*_*j*1_), (*e*_*i*2_, *e*_*j*2_),…, (*e*_*iN*_, *e*_*jN*_), and the* mean NND* is calculated. Assuming that $\overset{-}{d_{E}}$ is the mean of 100 randomly simulated mean NND and $\overset{-}{\sigma }$ is the standard variation, the *z*-score is calculated as

(5) $$\begin{array}{c} z=\frac{\overset{-}{d_{0}}-\overset{-}{d_{E}}}{\overset{-}{\sigma }}. \end{array}$$

We call the *z*-score as the* clustering index* for a scatter plot.

### 2.5. Layout for Visualization

Once the eigengenes with long tail or bimodal distributions are detected, SPLOM is generated. Each scatterplot is then colored using the color scale based on the clustering index. User can then select plots with interesting patters for further visualization and analysis.

## 3. Results

### 3.1. Datasets and Preprocessing

We applied the above analysis to two large gene expression single-cell datasets. One dataset is RNA sequencing data of single cells isolated from mouse dorsal lateral geniculate nucleus (dLGN) of the thalamus, which is downloaded from Allen Brian Atlas (ABA) website. This data set includes 1,772 single cells collected from dLGN in adult mouse and transcriptionally profiled with RNA sequencing. The dataset contains transcription readings for 45,772 genes and transcripts. However, since many of the genes have zero readings in most cells, these genes were filtered out; specifically we removed genes with zeros in more than half of the cells. In addition, genes whose mean values are among the lowest 20% and variances are among the lowest 50% were removed. This way, 20,000 genes were retained for further analysis.

Another dataset is from a single-cell study on human glioblastoma. The dataset was downloaded from NCBI Gene Expression Omnibus (GEO) with accession number GSE57872. It contains transcriptomes from 430 single glioblastoma cells isolated from 5 individual tumors and 102 single cells from gliomasphere cells lines generated using SMART-seq [27]. Using the same preprocessing procedure, 5,948 genes were kept for further analysis in this dataset.

### 3.2. Analysis of the ABA Mouse Brain scRNA-Seq Data

**Figure 3:**
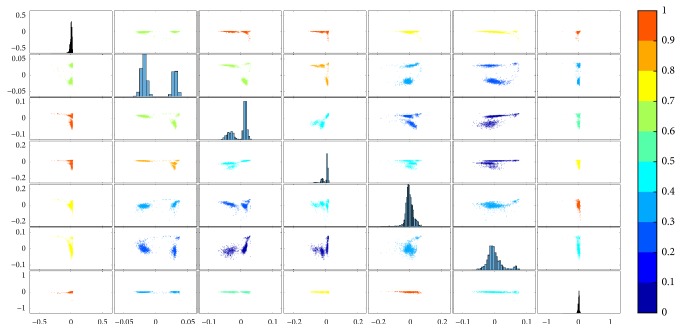
Colored SPLOM for the seven long tail eigengenes from the Allen Brain scRNA-seq data. The subplot in the *i*th row, *j*th column of the matrix is a scatter plot of the *i*th eigengene against the *j*th eigengene. Along the diagonal are histogram plots of each eigengene.

Using the lmQCM algorithm (with *γ* = 0.75), 60 coexpressed gene modules with at least five genes are identified. Using a threshold 20 for the Kurtosis metric, seven eigengenes are selected.  Table 1 summarizes the information for the seven modules.  Figure 3 shows the colored SPLOM for the seven eigengenes.

**Figure 4:**
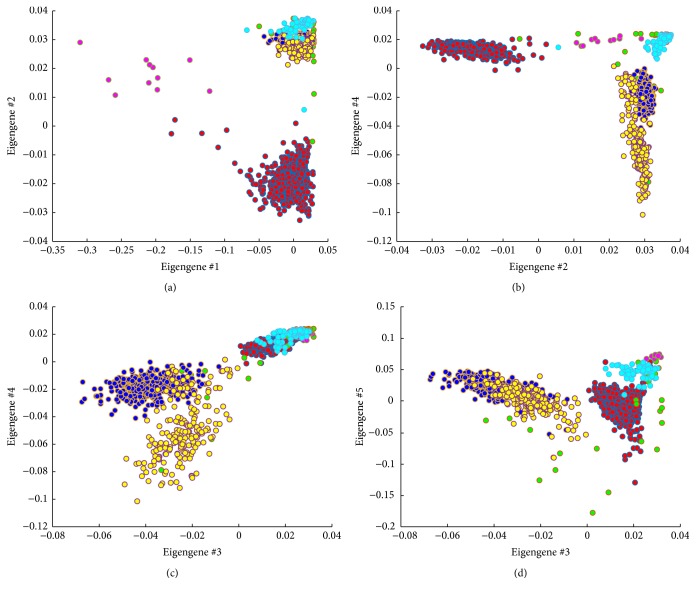
Four example scatterplots with broad classes annotated in different colors.

The color scheme in Figure 3 allows us to further inspect scatterplots with interesting patterns. In order to determine if these patterns are associated with specific annotations, for selected scatter plots, we further overlay the annotation information using different colors.  Figure 4 shows examples when the broad subtype information about the neurons is overlaid on the scatter plots as points with different colors. It is apparent that none of the gene modules can thoroughly separate the cells based on the subtypes. Instead, some of them can separate specific subtypes. For instance, as in Figure 4(a), the cells are separated into two major clusters based on the “clustering index” as defined in the previous section, which does not fully reflect the subtypes as the blue and yellow points are not separated. Instead, the blue and yellow points are segmented in Figure 4(b) and even further away in Figure 4(c).

As in Figure 4(b), it is clear that the groups of yellow cells and cyan cells are separated from the rest groups based on eigengene #4 that is enriched with genes that are important to bladder/pelvic ganglion development and may be involved in gender development too. At the same time, it can be noted that the red group is different from the blue, cyan, and yellow groups based on eigengene #2 that is closely associated with synapse formation. In addition, according to Figure 4(c), the blue and yellow groups are separated when both eigengenes #3 and #4 are involved and eigengene #3 is closely connected with the glutamate metabolism and inhibitory synapse development. These neural functions are critical for the interpretation of the cell population clustering.

**Figure 5:**
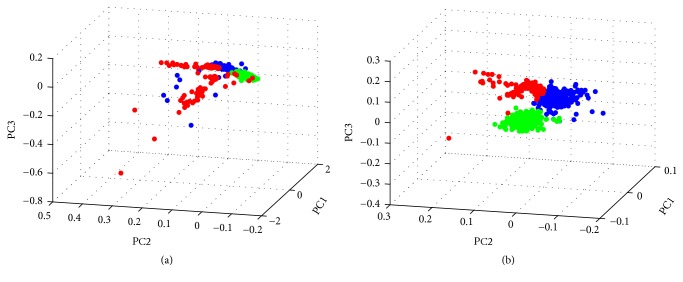
(a) The 3D plot for the first three principal components using all genes for the cells. (b) The 3D plot for the first three principal components using genes in the gene modules in Table 1 for the cells.

It is important to notice that the visual outcome is very different from traditional PCA based visualization. As shown in Figure 5(a), if all the genes are used for visualization of the cells using traditional PCA, there is not a clear separation of the cells except for a small group. If we limit the gene features for PCA to the ones involved only in the gene modules listed in Table 1, we can clearly see three major groups. As a control, we marked the three groups of cells in Figure 5(a) with three different colors, and we can see that there is no clear separation of the cells in Figure 5(a). However, without explicit functional grouping, it is difficult to determine which biological processes and functions are involved in such separation.

### 3.3. Analysis of the Human Glioblastoma Patients' Brain scRNA-Seq Data

Using the lmQCM algorithm (with *γ* = 0.2), 18 coexpressed gene modules with at least five genes are identified. Using a threshold of 5 for the Kurtosis metric, 16 eigengenes are selected.

**Figure 6:**
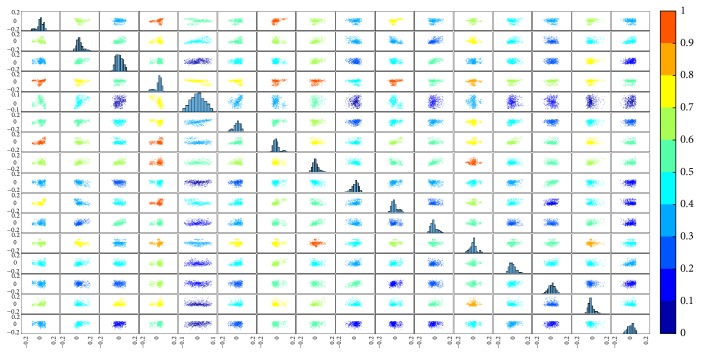
Colored SPLOM for the long tail eigengenes from the brain tumor study. The subplot in the *i*th row, *j*th column of the matrix is a scatter plot of the *i*th eigengene against the *j*th eigengene. Along the diagonal are histogram plots of each eigengene.

Figure 6 is the SPLOM for the long tail eigengenes from the brain tumor study.

**Figure 7:**
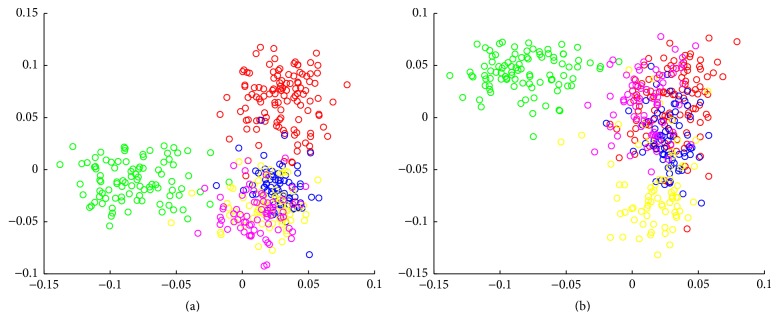
(a) The scatter plot between eigengene #4 (*x*-axis) and eigengene #11 (*y*-axis). (b) The scatter plot between eigengenes #4 (*x*-axis) and #6 (*y*-axis).

From the SPLOM, it is notable that the fourth gene module not only has an eigengene with bimodal distribution but also is involved in effective separation of the cells. While the cells are labeled by the patient and sample IDs, it is clear that some of the separation cases are closely related to the differences between different tumor samples as shown in Figure 7. In particular, eigengene #4 is key in separating the cells in the green group from the rest while other eigengenes can separate other groups (e.g., eigengene #6 separates the yellow cell group from the rest while eigengene #11 separates the red cell group). Interestingly enrichment analysis shows that this gene module for eigengene #4 is highly enriched with extracellular matrix genes (14 genes out of 36, *p* = 6.304*e* − 8) and the cell migration process (10 genes, *p* = 9.145*e* − 5), suggesting a particular property of the cells in the green group and it is important as the extracellular matrix and cell migration process is considered critical to the invasion of glioblastoma [28, 29].

## 4. Discussion and Conclusion

In this work, we presented a workflow for detecting distribution patterns in cell populations based on single-cell transcriptome study. With the fast adoption of single-cell analysis, a challenge to researchers is how to effectively extract gene features to meaningfully separate the cell population. However, this often ends up in a chicken-and-egg situation as the separation of the cells often depends on the choice of gene features, yet without a clear pattern it is difficult to determine which gene features are effective. Our workflow uses the well-developed gene coexpression network analysis to take advantage of the fact that coexpressed genes are often functionally or structurally related and the number of coexpressed modules is much smaller than the number of genes. Thus, when the coexpressed modules are summarized into eigengenes, not only can we quickly explore the distribution of cells interactively but also we can promptly interpret the gene features and generate new hypothesis.

Since the cells are separated based on different choices of the gene features, we dub the workflow as “functional virtual flow cytometry,” which achieves separation of the cells based on salient gene features. The separation of cells leads to new hypothesis such as the involvement of glutamate metabolism in the separation of the brain cells in the Allen Brain scRNA-seq data and the specific glioblastoma sample with unique cell migration related signature. While for the latter it is unclear if this observation is indeed biological or due to batch effect, our workflow quickly pointed out the pattern for researchers in deeper examination.

With the interactive visualization, additional advanced analysis can be carried out. For instance, in both Figures 4(b) and 7(b), an interesting observation is that the *x*- and *y*-axes cannot both have low values, suggesting interesting Boolean relationships between the gene groups [30]. Therefore, as our ongoing work, these analytic tools along with the workflow are being implemented in an online single-cell analytics portal.

## Figures and Tables

**Table 1:** The seven gene modules whose eigengenes show long tail distributions.

Eigengene #	Index	Size	Kurtosis	Enrichment/notes
1	3	38	10.7844	32 predicted genes: three genes are immunoglobulins and two are T cell receptors, acute lymphocytic leukemia (*p* = 3.157*e* − 7)

2	6	35	5.0379	Ion transport (*p* = 3.341*e* − 7), synapse (*p* = 2.590*e* − 7)

3	12	18	8.5550	Glutamate decarboxylation to succinate (*p* = 7.715*e* − 7), inhibitory synapse (*p* = 7.843*e* − 7)

4	13	17	19.9492	Development of lower uro neuro e15.5 BladdPelvicGanglion Sox10 top-relative-expression-ranked 1000 (1.227*e* − 7), six genes on chromosome X

5	28	11	4.9068	Hydrogen ion transmembrane transport (*p* = 4.859*e* − 20), mitochondrial inner membrane (*p* = 1.533*e* − 16)

6	48	6	3.8686	NADH metabolic process (*p* = 2.960*e* − 13), myelin sheath (*p* = 1.643*e* − 3), gluconeogenesis (*p* = 5.401*e* − 14), genes upregulated in hippocampus at late postnatal stages (*p* = 9.341*e* − 10)

7	60	5	12.5680	Mostly predicted genes

## Abbreviations

SPLOM: Scatter plot matrix
scRNA-seq: Single-cell RNA sequencing
PCA: Principal component analysis
MDS: Multidimensional scaling
FVFC: Functional virtual flow cytometry
GCNA: Gene coexpression network analysis
dLGN: Dorsal lateral geniculate nucleus
ABA: Allen Brian Atlas
GEO: Gene Expression Omnibus.
