# Corrigendum to “Functional Virtual Flow Cytometry: A Visual Analytic Approach for Characterizing Single-Cell Gene Expression Patterns”

**DOI:** 10.1155/2017/9393251

**Published:** 2017-11-13

**Authors:** Zhi Han, Travis Johnson, Jie Zhang, Xuan Zhang, Kun Huang

**Affiliations:** ^1^College of Software, Nankai University, Tianjin, China; ^2^Department of Biomedical Informatics, The Ohio State University, Columbus, OH, USA; ^3^The CCC Biomedical Informatics Shared Resource, The Ohio State University, Columbus, OH, USA

In the article titled “Functional Virtual Flow Cytometry: A Visual Analytic Approach for Characterizing Single-Cell Gene Expression Patterns” [1], an acknowledgment should be added as follows:

The work was also partially supported by the Shenzhen Peacock Plan (KQTD2016053112051497 to Kun Huang).
